# The role of psychological flexibility in the relationship between empathy and flourishing among clinical nurses: a cross-sectional mediation analysis

**DOI:** 10.3389/fpsyg.2026.1923032

**Published:** 2026-09-03

**Authors:** Xiangjun Guo, Li Liu, Hangqin Lv, Meichuan Lin, Liping Chen, Hanting Zhang, Menghan Liu, Yutong Liu, Wenyan Jiang, Xiaoyun Liu

**Affiliations:** 1 School of Preclinical Medicine and School of Nursing, Chengdu University, Chengdu, Sichuan, China; 2 Chengdu Industry & Trade College, Chengdu, China

**Keywords:** clinical nurses, empathy, flourishing, mediation analysis, psychological flexibility

## Abstract

**Background:**

Clinical empathy is essential for high-quality patient care; however, its association with nurses' positive psychological functioning remains insufficiently understood. Psychological flexibility may be relevant to this association because it reflects how nurses respond to the difficult internal experiences that may arise during emotionally demanding care. This study examined the cross-sectional associations among clinical empathy, psychological flexibility, and flourishing in clinical nurses and evaluated the indirect association through psychological flexibility.

**Methods:**

A cross-sectional study was conducted among 545 clinical nurses recruited from three tertiary Grade A hospitals in Sichuan Province, China, in May 2026. Self-reported data were collected on clinical empathy, psychological flexibility, flourishing, and sociodemographic and work-related characteristics. Pearson correlation analysis, hierarchical multiple linear regression, and statistical mediation analysis using the SPSS PROCESS macro (Model 4) were conducted. The indirect association was evaluated using 5,000 bootstrap resamples with percentile bootstrap confidence intervals.

**Results:**

The mean flourishing score was 46.13 (SD = 8.21). Flourishing was positively correlated with empathy (*r* = 0.157, *p* < 0.001) and psychological flexibility (*r* = 0.662, *p* < 0.001), whereas empathy was weakly correlated with psychological flexibility (*r* = 0.129, *p* = 0.002). In the final regression model, psychological flexibility showed a strong positive association with flourishing (β = 0.627, *p* < 0.001), whereas the adjusted direct association between empathy and flourishing was small (β = 0.066, *p* = 0.034). The estimated indirect association through psychological flexibility was 0.046 (BootSE = 0.016, 95% percentile bootstrap CI [0.013, 0.078]). In sensitivity analyses, the indirect association remained statistically significant across different covariate specifications, although its magnitude was attenuated after concurrently measured job satisfaction was included.

**Conclusions:**

Psychological flexibility was more strongly and consistently associated with flourishing than empathy. The significant indirect association through psychological flexibility was consistent with the hypothesized model but does not establish temporal or causal mediation because all variables were measured concurrently. Longitudinal and intervention studies are needed to clarify the temporal ordering of these constructs and to determine whether changes in psychological flexibility precede changes in flourishing.

## 1 Introduction

Nurses are an essential component of healthcare systems. Their physical and mental wellbeing is closely related not only to their quality of life but also to the quality of nursing care, patient safety, and workforce stability (Ruiz-Fernández et al., 2020). However, clinical nursing is often characterized by heavy workloads, complex and dynamic clinical situations, and sustained emotional labor, placing nurses at increased risk of psychological stress, distress, and burnout (Baek and Cha, 2025; Roelen et al., 2018). With the development of positive psychology, mental health is no longer regarded merely as the absence of psychological problems but also as the presence of positive psychological functioning (Lai et al., 2022; Seligman and Csikszentmihalyi, 2000). Flourishing, an important concept in positive psychology, reflects an individual's overall wellbeing and positive functioning across emotional, psychological, and social domains (Keyes, 2007). Previous studies have shown that higher levels of flourishing are associated with lower negative affect, greater life satisfaction, and better overall quality of life (Amonoo et al., 2023). Therefore, identifying psychological and contextual factors associated with nurses' flourishing may contribute to a more comprehensive understanding of their occupational wellbeing.

Empathy is an important competency that enables clinical nurses to establish effective nurse–patient relationships and deliver patient-centered care (Brunero et al., 2010; van Dijke et al., 2020). Previous studies have mainly focused on the beneficial effects of empathy on patient outcomes, including improved patient satisfaction, treatment adherence, and nurse–patient communication (Howick et al., 2020; Keshtkar et al., 2024; Cai et al., 2026). Empathy may also have important psychological implications for nurses themselves. On the one hand, by understanding and responding to patients' pain, fear, and emotional needs, nurses may experience greater professional meaning, compassion satisfaction, and personal growth during care provision (Zeng et al., 2024). On the other hand, sustained exposure to patients' negative emotions and suffering may increase nurses' emotional burden and may be associated with compassion fatigue and emotional exhaustion (Sacco et al., 2015; Figley, 1995; Gustafsson and Hemberg, 2022). These findings suggest that the association between empathy and nurses' positive psychological functioning may depend partly on how nurses respond to the emotional experiences encountered during empathic care.

### 1.1 Theoretical framework and hypotheses

The psychological flexibility model of Acceptance and Commitment Therapy (ACT) provides a theoretical perspective for understanding this process (Wersebe et al., 2018). Psychological flexibility refers to the capacity to remain aware of and open to one's thoughts and emotions and to continue acting in accordance with personally meaningful values in complex situations (Hayes et al., 2006). It comprises six interrelated core processes: acceptance, cognitive defusion, present moment awareness, self-as-context, values, and committed action. Rather than primarily seeking to eliminate distressing experiences, these processes help individuals choose actions flexibly in accordance with their goals and values even in the presence of difficult internal experiences, thereby supporting adaptive functioning and positive mental health (Zarvijani et al., 2021; Kashdan and Rottenberg, 2010; Chong et al., 2023).

Empathic nursing care requires nurses to understand patients' perspectives and emotions while remaining aware of their own emotional reactions, maintaining an appropriate distinction between their own experiences and those of patients, and continuing to respond in accordance with professional values (Xu et al., 2023; Riess, 2017; Juniarta Eka and Ferawati Sitanggang, 2024). These requirements are conceptually related to present moment awareness, acceptance, cognitive defusion, self-as-context, and value-guided action within the psychological flexibility model. Thus, although empathy and psychological flexibility are distinct psychological constructs, they may be positively associated.

The conceptual links described above not only suggest a possible association between empathy and psychological flexibility but also provide a basis for understanding how psychological flexibility may be involved in the association between empathy and flourishing. Empathic care is often accompanied by complex internal experiences, and how nurses respond to these experiences may be related to their positive psychological functioning. Psychological flexibility may help nurses remain open to and accept these internal experiences while continuing to act in accordance with professional values. It may therefore be positively associated with flourishing and statistically account for part of the association between empathy and flourishing.

**Figure 1:**
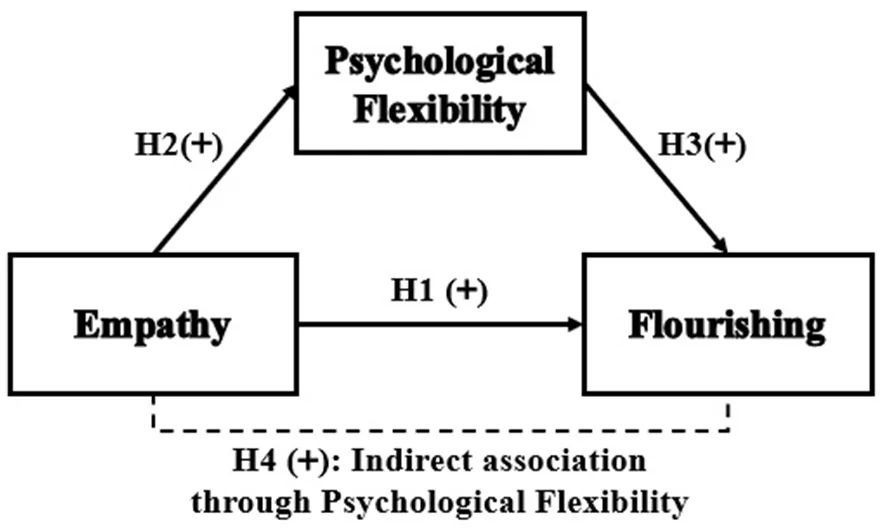
Hypothesized model linking empathy, psychological flexibility, and flourishing. Solid arrows represent the hypothesized positive associations among empathy, psychological flexibility, and flourishing. The dashed bracket represents the hypothesized indirect association between empathy and flourishing through psychological flexibility.

In summary, although previous studies have separately examined empathy or psychological flexibility in relation to nurses' mental health, integrative research on the relationships among empathy, psychological flexibility, and flourishing remains limited. The present study developed a hypothesized model linking empathy, psychological flexibility, and flourishing (Figure 1). It aimed to examine the associations of empathy with psychological flexibility and flourishing and to determine whether psychological flexibility statistically accounted for part of the association between empathy and flourishing. Accordingly, the following hypotheses were proposed:

H1: Empathy is positively associated with flourishing.
H2: Empathy is positively associated with psychological flexibility.
H3: Psychological flexibility is positively associated with flourishing.
H4: Empathy is indirectly associated with flourishing through psychological flexibility.

## 2 Materials and methods

### 2.1 Study design and participants

This cross-sectional study was reported in accordance with the Strengthening the Reporting of Observational Studies in Epidemiology (STROBE) guideline for cross-sectional studies. Using convenience sampling, participants were recruited from three tertiary Grade A hospitals in Sichuan Province, China. Data were collected in May 2026. The inclusion criteria for the participants were as follows: (1) holding a valid registered nurse (RN) license; (2) currently working in clinical nursing or nursing-related positions, including staff nurses, nursing educators, and nurse managers, in a tertiary Grade A hospital; and (3) providing voluntary informed consent to participate in this study. The exclusion criteria included: (1) nursing students, interns, or visiting nurses; or (2) on long-term leave, including sick leave, maternity leave, or study leave, during the data collection period. An initial sample-size estimate was based on the commonly used rule of thumb of 5–10 respondents per questionnaire item (Stuart and Ord, 2010; Wang et al., 2024). As the questionnaire comprised 58 items, the minimum required sample size was 290 participants. To allow for an anticipated 20% rate of invalid or incomplete questionnaires, the target sample size was calculated as 290/ (1–0.20) = 362.5 and rounded up to 363 participants. A total of 545 valid questionnaires were included in the final analysis.

To provide a supplementary assessment of the statistical power for detecting the observed indirect association, a *post hoc* Monte Carlo analysis was conducted using the method described by Schoemann et al. (Schoemann et al., 2017). The analysis was based on the standardized coefficients estimated from the primary covariate-adjusted model (*a* = 0.124, *b* = 0.627, and *c*′ = 0.066), a sample size of 545, and a two-sided significance level of α = 0.05. Under the observed parameter estimates, the estimated power to detect an indirect association of this magnitude was 0.83, indicating a reasonable probability of detecting the observed indirect association with the final sample. This *post hoc* result was considered supplementary to the prespecified sample-size calculation.

### 2.2 Data collection and quality control

Data were collected anonymously through Wenjuanxing, an online survey platform in China. The survey link and QR code were distributed through nurses' WeChat work groups by nursing administrators or head nurses at the participating hospitals. Before accessing the questionnaire, participants were presented with an electronic informed consent form that explained the study purpose, the voluntary nature of participation, and the confidentiality of the collected data. Participants selected “I Agree” before proceeding to the questionnaire. Several approaches were used to help improve data completeness and quality. Except for items involving skip logic, applicable questions were set as mandatory to reduce missing responses. The survey system was set to limit repeated submissions from the same IP address and WeChat account. After data collection, responses were reviewed for completion time and obvious logical inconsistencies. Questionnaires completed in < 100 s or showing clear inconsistencies were considered for exclusion. The 100-s threshold corresponded to an average completion time of fewer than 2 s per item across the 58-item questionnaire and was considered insufficient for careful reading and responding (Xu et al., 2025; Bowling et al., 2023). A total of 569 questionnaires were initially collected. After review, 24 questionnaires were excluded because of potential data-quality concerns, resulting in a final sample of 545 valid questionnaires and an effective response rate of 95.8%.

### 2.3 Measures

#### 2.3.1 Sociodemographic questionnaire

Sociodemographic and work-related information was collected using a self-designed questionnaire. The questionnaire included gender, age, years of nursing experience, department, educational level, professional title, position, monthly income, employment type, daily working hours, weekly working days, night shifts per month, job satisfaction, marital status, marital satisfaction, number of children, only-child status, and parental health status.

#### 2.3.2 Flourishing scale

The positive psychological functioning and overall wellbeing of clinical nurses were assessed using the Flourishing Scale (FS). Originally developed by Diener et al. (2010), the present study utilized the Simplified Chinese version of the FS, which was translated and culturally adapted by Tang et al. (2016). This unidimensional scale comprises 8 items, primarily measuring individuals' self-perceived success in important areas such as relationships, self-esteem, purpose, and optimism (e.g., “I am engaged and interested in my daily activities”). The instrument employs a 7-point Likert scoring system ranging from one (strongly disagree) to seven (strongly agree), with all items being positively scored. The total score is obtained by summing the responses, yielding a possible range of eight to 56. Higher scores indicate higher levels of flourishing. The Chinese version of the FS has been used among nurses and demonstrated good internal consistency, with a reported Cronbach's α of 0.90 (Cui et al., 2026). In the present study, the Cronbach's α was 0.96, indicating excellent internal consistency.

#### 2.3.3 The Jefferson scale of empathy-health professionals (JSE-HP)

To evaluate the clinical empathy of the participating nurses, this study employed the version for health professionals of the Jefferson Scale of Empathy (JSE-HP), originally developed by Hojat et al. (2001). The JSE-HP is a widely used instrument for assessing empathy among healthcare professionals. The scale consists of 20 items rated on a 7-point Likert scale ranging from one (strongly disagree) to seven (strongly agree). Total scores range from 20 to 140, with higher scores indicating greater levels of clinical empathy. The Chinese version developed by Ma Li was used in the present study (Ma, 2009). In the original Chinese validation study, the total scale demonstrated acceptable internal consistency, with a Cronbach's α of 0.80. The JSE-HP was used in this study with permission from Thomas Jefferson University. In the present study, the scale demonstrated good internal consistency, with a Cronbach's α of 0.84.

#### 2.3.4 Psychological flexibility subscale of the simplified multidimensional psychological flexibility inventory (MPFI-24)

Psychological flexibility among clinical nurses was assessed using the Psychological Flexibility subscale of the Simplified Multidimensional Psychological Flexibility Inventory (MPFI-24). The original MPFI was developed by Rolffs et al. (2018) based on Acceptance and Commitment Therapy (ACT), and the present study employed the Chinese version translated and culturally adapted by Liu et al. (2023). The subscale has also been used among nurses, with a reported Cronbach's α of 0.90 (Sun et al., 2025). This 12-item subscale comprises six dimensions: acceptance, cognitive defusion, present moment awareness, self-as-context, values, and committed action. Respondents were asked to report their actual experiences over the past 2 weeks. Each item is rated on a 6-point Likert scale ranging from one (never true) to six (always true). The total score ranges from 12 to 72, with higher scores indicating greater psychological flexibility. In the present study, Cronbach's α was 0.95, indicating excellent internal consistency.

### 2.4 Data analysis

#### 2.4.1 Descriptive and preliminary analyses

All statistical analyses were performed using IBM SPSS Statistics version 26.0 (IBM Corp., Armonk, NY, USA). Continuous variables were summarized using means and standard deviations, whereas categorical variables were described using frequencies and percentages. Independent-samples t tests and one-way analyses of variance were used to compare flourishing scores across participant characteristics. Pearson correlation analysis was conducted to examine the bivariate associations among empathy, psychological flexibility, and flourishing. Harman's single-factor test was performed as a preliminary assessment of common method variance by entering all items measuring empathy, psychological flexibility, and flourishing into an unrotated factor analysis.

#### 2.4.2 Hierarchical regression and indirect-association analysis

Hierarchical multiple linear regression was used to examine the independent associations of empathy and psychological flexibility with flourishing and to assess their incremental contributions to the explained variance beyond the covariates. Covariates were selected by considering previous evidence, clinical relevance, and their observed associations with flourishing in the preliminary analyses. The primary model included age, gender, daily working hours, night shifts per month, marital status, and parental health status, representing demographic, work-demand, and family-context factors. These covariates were entered in Model 1, empathy was added in Model 2, and psychological flexibility was added in Model 3. Age, daily working hours, and night shifts per month were entered using their original continuous values. Nominal categorical variables were represented using dummy variables, with male gender, married status, and healthy parental status as the reference categories. Multicollinearity was assessed using variance inflation factors, and first-order autocorrelation of residuals was examined using the Durbin–Watson statistic.

The hypothesized indirect association was examined using the PROCESS macro for SPSS version 4.2. Model 4 was selected because the proposed conceptual model specified a simple single-mediator structure comprising one independent variable (empathy), one statistical mediator (psychological flexibility), and one dependent variable (flourishing), without moderation or serial mediation. Path a represented the association between empathy and psychological flexibility. Path *b* represented the association between psychological flexibility and flourishing after adjustment for empathy. Path *c* represented the total association between empathy and flourishing before psychological flexibility was included in the outcome model, whereas path *c*′ represented the direct association between empathy and flourishing after psychological flexibility was included. The indirect association through psychological flexibility was estimated as the product of paths *a* and *b* (*a* × *b*). The same covariates used in the primary regression analysis were included in both the mediator and outcome models.

The indirect association was evaluated using 5,000 percentile bootstrap resamples. Bootstrapping was used to construct an empirical sampling distribution of the product term *a* × *b* and does not assume that the sampling distribution of the indirect-association estimate is normal. This approach is appropriate because the distribution of a product of coefficients may be asymmetric. The use of 5,000 resamples provided a stable basis for estimating the percentile bootstrap confidence interval. An indirect association was considered statistically significant when its 95% percentile bootstrap confidence interval did not include zero. Given the cross-sectional design, paths *a, b, c*, and *c*′, as well as the indirect association, were interpreted as statistical associations among concurrently measured variables rather than as evidence of temporal or causal mediation.

#### 2.4.3 Sensitivity analyses

Sensitivity analyses were conducted to examine the stability of the findings under broader covariate adjustment. The expanded model additionally included department, weekly working days, professional title, position, and having children. Weekly working days were entered using the original continuous value. Department and position were represented using dummy variables, with internal medicine and staff nurse as the respective reference categories.

Because of the small numbers of participants in the higher professional-title categories, professional title was regrouped as junior, intermediate, and senior. The junior category included nurses and senior nurses, the intermediate category included nurses-in-charge, and the senior category included associate chief nurses and chief nurses. Junior professional title was used as the reference category. Having children was derived from the reported number of children and coded as no children or having at least one child, with no children as the reference category.

In a further exploratory sensitivity analysis, concurrently measured job satisfaction was added to the expanded model using its original five-point ordered score, with higher scores indicating greater job satisfaction. Hierarchical regression and PROCESS Model 4 analyses were repeated under each covariate specification. All statistical tests were two-sided, and *p* < 0.05 was considered statistically significant.

### 2.5 Ethical considerations

In this study, electronic informed consent was obtained from all participants, and ethical approval was granted by the Medical Ethics Committee of the Affiliated Hospital of Chengdu University (PJ2025-142-03).

## 3 Results

### 3.1 Common method variance

An unrotated exploratory factor analysis of the items measuring empathy, psychological flexibility, and flourishing identified six factors with eigenvalues >1. The first factor accounted for 30.81% of the total variance. This result did not indicate that a single factor dominated the item covariance; however, Harman's single-factor test cannot rule out common method variance, and the result was interpreted only as a preliminary diagnostic.

### 3.2 Participant characteristics and group comparisons in flourishing

A total of 545 clinical nurses were included in the analysis. The mean age of the participants was 34.39 years (SD = 7.31), and the mean duration of nursing experience was 12.80 years (SD = 8.24). As shown in Table 1, most participants were female (92.7%), married (72.1%), and held a bachelor's degree (87.3%). Nearly half of the participants were aged 26–35 years (49.4%), and 43.5% had 11–20 years of nursing experience. The mean flourishing score was 46.13 (SD = 8.21). Group comparisons showed statistically significant differences in flourishing by age, department, daily working hours, weekly working days, professional title, position, night shifts per month, job satisfaction, marital status, marital satisfaction, number of children, and parental health status. The difference according to monthly income did not meet the prespecified threshold for statistical significance (*p* =0.050). No statistically significant differences in flourishing were observed according to gender, years of nursing experience, education level, employment type, or only-child status (all *p* > 0.05; Table 1).

**Table 1:** Comparison of flourishing scores by participant characteristics (*N* = 545).

Variables	Group	*N* (%)	Mean ±SD	*t/F*	*p*
Gender	Male	40 (7.3%)	46.10 ± 8.13	−0.021	0.983
	Female	505 (92.7%)	46.13 ± 8.23		
Age	≤ 25	50 (9.2%)	44.00 ± 9.01	3.536	0.015
	26–35	269 (49.4%)	45.91 ± 8.54		
	36–45	173 (31.7%)	46.17 ± 7.53		
	>45	53 (9.7%)	49.09 ± 7.24		
Nursing experience (years)	≤ 5	103 (18.9%)	45.28 ± 8.02	2.557	0.054
	6–10	127 (23.3%)	45.30 ± 8.97		
	11–20	237 (43.5%)	46.24 ± 7.94		
	>20	78 (14.3%)	48.24 ± 7.72		
Department	Outpatient	49 (9.0%)	48.06 ± 8.39	2.554	0.038
	Internal medicine	187 (34.3%)	45.12 ± 8.34		
	Surgery	159 (29.2%)	45.66 ± 8.44		
	Emergency/ICU	43 (7.9%)	46.02 ± 7.74		
	Other departments	107 (19.6%)	47.73 ± 7.49		
Daily working hours (h)	≤ 8	386 (70.8%)	46.84 ± 7.91	5.233	0.006
	8–10	133 (24.4%)	44.51 ± 8.54		
	>10	26 (4.8%)	43.73 ± 9.53		
Weekly working days (days)	≤ 5	523 (96.0%)	46.29 ± 8.15	2.309	0.021
	>5	22 (4.0%)	42.18 ± 8.82		
Education level	Junior college	54 (9.9%)	46.93 ± 8.55	0.283	0.753
	Bachelor's degree	476 (87.3%)	46.04 ± 8.21		
	Master's degree or above	15 (2.8%)	46.00 ± 7.36		
Professional title	Nurse	55 (10.1%)	43.58 ± 9.73	2.474	0.043
	Senior nurse	190 (34.9%)	45.55 ± 8.53		
	Nurse-in-charge	268 (49.2%)	46.84 ± 7.76		
	Associate chief nurse	27 (5.0%)	47.78 ± 5.28		
	Chief nurse	5 (0.9%)	48.60 ± 9.56		
Position	Staff nurse	420 (77.1%)	45.72 ± 8.46	3.980	0.019
	Nursing educator	85 (15.6%)	46.54 ± 7.64		
	Nurse manager	40 (7.3%)	49.48 ± 5.68		
Monthly income	1,500–3,000	20 (3.7%)	46.75 ± 8.58	2.615	0.050
	3,001–6,000	147 (27.0%)	44.59 ± 8.60		
	6,001–9,500	273 (50.1%)	46.48 ± 7.94		
	>9,500	105 (19.3%)	47.26 ± 8.11		
Night shifts per month	0	143 (26.2%)	47.41 ± 7.84	3.591	0.014
	1–5	170 (31.2%)	46.78 ± 7.46		
	6–10	186 (34.1%)	44.66 ± 8.98		
	>10	46 (8.4%)	45.70 ± 8.09		
Employment type	Contract-based	467 (85.7%)	46.02 ± 8.36	−0.807	0.421
	Permanent	78 (14.3%)	46.76 ± 7.28		
Job satisfaction	Strongly dislike	6 (1.1%)	44.00 ± 12.39	54.345	< 0.001
	Slightly dislike	13 (2.4%)	39.31 ± 11.48		
	Neutral	173 (31.7%)	40.80 ± 7.67		
	Like	256 (47%)	47.54 ± 6.49		
	Strongly like	97 (17.8%)	52.93 ± 5.54		
Marital status	Unmarried	137 (25.1%)	44.83 ± 8.72	8.070	< 0.001
	Married	393 (72.1%)	46.83 ± 7.76		
	Divorced	15 (2.8%)	39.60 ± 11.04		
Marital satisfaction^**a**^	Very dissatisfied	1 (0.2%)	37.00	29.203	< 0.001
	Dissatisfied	7 (1.7%)	32.57 ± 10.61		
	Neutral	54 (13.2%)	39.96 ± 8.02		
	Satisfied	188 (46.1%)	46.04 ± 6.94		
	Very satisfied	158 (38.7%)	50.11 ± 6.74		
Number of children	0	197 (36.1%)	44.89 ± 8.89	2.878	0.036
	1	263 (48.3%)	46.57 ± 7.92		
	2	83 (15.2%)	47.49 ± 7.15		
	>2	2 (0.4%)	52.00 ± 5.66		
Only-child status	No	322 (59.1%)	46.00 ± 8.06	−0.432	0.666
	Yes	223 (40.9%)	46.31 ± 8.44		
Parental health Status	Chronic/major Illness	138 (25.3%)	44.68 ± 9.30	6.510	< 0.001
	Suboptimal health	160 (29.4%)	45.26 ± 7.84		
	Healthy	235 (43.1%)	47.79 ± 7.24		
	Other	12 (2.2%)	41.83 ± 11.80		

Data are presented as *n* (%) or mean ± SD. Independent-samples t tests were used for comparisons between two groups, and one-way analysis of variance was used for comparisons among three or more groups. ICU, intensive care unit; SD, standard deviation.

^a^Data on marital satisfaction were collected only from participants who were married or divorced (*n* = 408).

### 3.3 Descriptive statistics and correlations among study variables

As shown in Table 2, the mean scores for flourishing, empathy, and psychological flexibility were 46.13 (SD = 8.21), 101.18 (SD = 13.79), and 55.32 (SD = 10.97), respectively. Pearson correlation analysis showed that flourishing was significantly and positively correlated with psychological flexibility (*r* = 0.662, *p* < 0.001) and empathy (*r* = 0.157, *p* < 0.001). Empathy was also weakly but significantly positively correlated with psychological flexibility (*r*=0.129, *p* = 0.002).

**Table 2:** Descriptive statistics and correlations among the study variables (*N* = 545).

Variables	Mean ±SD	1	2	3
1. Flourishing	46.13 ± 8.21	1		
2. Empathy	101.18 ± 13.79	0.157^**^	1	
3. Psychological flexibility	55.32 ± 10.97	0.662^**^	0.129^**^	1

^**^p < 0.01(two-tailed).

### 3.4 Factors associated with flourishing

Hierarchical multiple linear regression analysis was conducted to examine factors associated with flourishing among clinical nurses. Age, gender, daily working hours, night shifts per month, marital status, and parental health status were entered in Model 1. Empathy was added in Model 2, and psychological flexibility was further added in Model 3. No serious multicollinearity was detected, with all variance inflation factors below 5.

As shown in Table 3, Model 1 explained 10.2% of the variance in flourishing (*R*^2^ = 0.102, adjusted *R*^2^ = 0.087, *p* < 0.001). After empathy was added in Model 2, the explained variance increased significantly to 12.2% (ΔR^2^ = 0.020, *p* < 0.001). After psychological flexibility was further added in Model 3, the final model explained 50.0% of the variance in flourishing (*R*^2^ = 0.500, adjusted *R*^2^ = 0.489, ΔR^2^ = 0.377, *p* < 0.001).

**Table 3:** Hierarchical Multiple Linear Regression Analysis of Variables Associated with Flourishing (*N* = 545).

Variables	Model 1 *B* (SE)	β	*p*	Model 2 *B* (SE)	β	*p*	Model 3 *B* (SE)	β	*p*
Age	0.142 (0.061)	0.127	0.020	0.135 (0.061)	0.120	0.026	0.163 (0.046)	0.145	<0.001
Daily working hours (h)	−1.057 (0.352)	−0.125	0.003	−0.973 (0.349)	−0.115	0.006	−0.603 (0.264)	−0.071	0.023
night shifts per month	−0.106 (0.090)	−0.054	0.238	−0.117 (0.089)	−0.059	0.189	−0.020 (0.067)	−0.010	0.764
Female^a^	−0.673 (1.304)	−0.021	0.606	−0.853 (1.292)	−0.027	0.509	−0.868 (0.976)	−0.028	0.374
Marital status: unmarried^b^	−0.833 (0.931)	−0.044	0.371	−0.671 (0.922)	−0.035	0.467	0.148 (0.698)	0.008	0.832
Marital status: divorced^b^	−7.762 (2.082)	−0.155	<0.001	−7.613 (2.061)	−0.152	<0.001	−5.932 (1.560)	−0.118	<0.001
Parental health status:chronic/major illness^c^	−3.667 (0.860)	−0.194	<0.001	−3.601 (0.851)	−0.191	<0.001	−2.732 (0.645)	−0.145	<0.001
Parental health status:suboptimal health^c^	−2.143 (0.808)	−0.119	0.008	−2.310 (0.801)	−0.128	0.004	−1.463 (0.607)	−0.081	0.016
Parental health status: other^c^	−8.163 (2.373)	−0.146	0.001	−8.152 (2.348)	−0.146	0.001	−7.564 (1.775)	−0.135	<0.001
Empathy	–	–	–	0.086 (0.024)	0.144	<0.001	0.039 (0.019)	0.066	0.034
Psychological flexibility	–	–	–	–	–	–	0.470 (0.023)	0.627	<0.001
*R* ^2^	0.102			0.122			0.500		
Adjusted *R*^2^	0.087			0.106			0.489		
Δ*R*^2^	0.102			0.020			0.377		
*F*	6.760		<0.001	7.449		<0.001	48.387		<0.001

*B*, unstandardized regression coefficient; SE, standard error; β, standardized regression coefficient. Model 1 included age, gender, daily working hours, night shifts per month, marital status, and parental health status. Model 2 added empathy, and Model 3 further added psychological flexibility. Age, daily working hours, and night shifts per month were entered as continuous variables using their original values. Gender, marital status, and parental health status were entered as dummy variables. The Durbin–Watson statistic for the final model was 2.157. All variance inflation factors were below 5, indicating no serious multicollinearity.

^a^Reference group = Male.

^b^Reference group = Married.

^c^Reference group = Healthy.

In the final model, psychological flexibility showed a strong positive association with flourishing (*B* = 0.470, β = 0.627, *p* < 0.001). Empathy retained a statistically significant but small positive association with flourishing (*B* = 0.039, β = 0.066, *p* = 0.034). In addition, older age was positively associated with flourishing, whereas longer daily working hours, divorced marital status, and poorer parental health status were negatively associated with flourishing (all *p* < 0.05).

### 3.5 Statistical mediation analysis

A statistical mediation analysis was conducted using PROCESS Model 4 to examine whether psychological flexibility accounted for part of the cross-sectional association between empathy and flourishing. Age, gender, daily working hours, night shifts per month, marital status, and parental health status were included as covariates.

**Figure 2:**
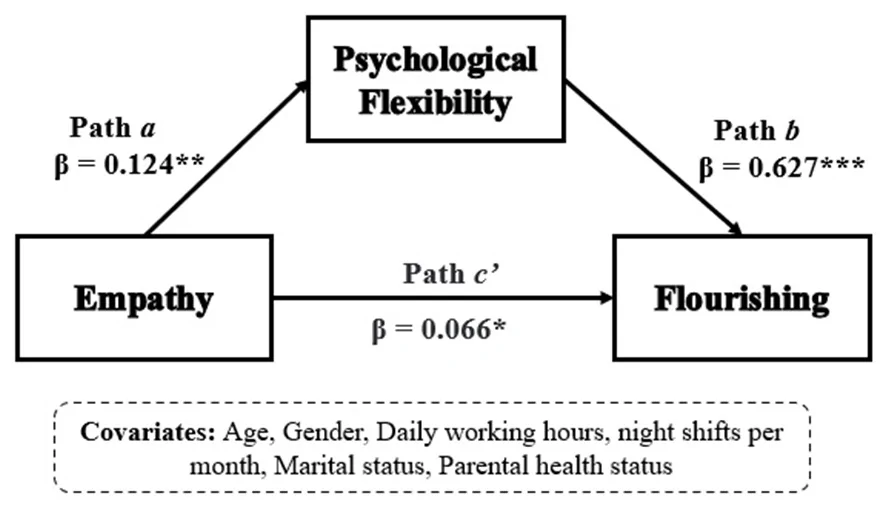
Estimated indirect-association model linking empathy, psychological flexibility, and flourishing in the primary model. Standardized coefficients are presented. Path a represents the association between empathy and psychological flexibility; path *b* represents the association between psychological flexibility and flourishing after adjustment for empathy; and path *c*′ represents the direct association between empathy and flourishing after psychological flexibility was included in the model. The primary model adjusted for age, gender, daily working hours, night shifts per month, marital status, and parental health status. *p < 0.05, **p < 0.01, ***p < 0.001.

Empathy was positively associated with psychological flexibility (*B* = 0.098, SE = 0.034, β = 0.124, *p* = 0.004), whereas psychological flexibility was strongly associated with flourishing after adjustment for empathy and the covariates (*B* = 0.470, SE = 0.023, β = 0.627, *p* < 0.001). The total association between empathy and flourishing was statistically significant but modest (*B* = 0.086, SE = 0.024, β = 0.144, *p* < 0.001). The estimated indirect association through psychological flexibility was 0.046 (BootSE = 0.016), and the 95% percentile bootstrap confidence interval did not include zero (0.013–0.078). The completely standardized indirect association was 0.078 (95% percentile bootstrap CI [0.022, 0.131]). The direct association between empathy and flourishing was small but statistically significant in the primary model (*B* = 0.039, SE = 0.019, β = 0.066, *p* = 0.034). Detailed estimates for the primary model are presented in Table 4 and Figure 2.

**Table 4:** Estimated total, direct, and indirect associations between empathy and flourishing through psychological flexibility in the primary model.

Association	*B*	SE/BootSE	β	*t*	*p*	95%CI
Total association, path *c* (Empathy → Flourishing)	0.086	0.024	0.144	3.515	<0.001	[0.038, 0.134]
Direct association, path *c*′(Empathy → Flourishing)	0.039	0.019	0.066	2.126	0.034	[0.003, 0.076]
Indirect association (Empathy → Psychological Flexibility → Flourishing)	0.046	0.016	0.078	–	–	[0.013, 0.078]

*B*, unstandardized association estimate; SE, standard error; BootSE, bootstrap standard error; β, standardized coefficient or completely standardized indirect association; CI, confidence interval. Path *c* represents the total association between empathy and flourishing, whereas path *c*′ represents the direct association between empathy and flourishing after psychological flexibility was included in the model. The analysis was adjusted for age, gender, daily working hours, night shifts per month, marital status, and parental health status. Age, daily working hours, and night shifts per month were entered as continuous variables using their original values. Gender, marital status, and parental health status were entered as dummy variables. For the indirect association, the value in the SE/BootSE column represents the bootstrap standard error, β represents the completely standardized indirect association, and the 95% CI corresponds to the unstandardized indirect association. The indirect association was estimated using 5,000 percentile bootstrap samples. The indirect association was considered statistically significant when the 95% percentile bootstrap confidence interval did not include zero.

### 3.6 Sensitivity analyses

Two sensitivity analyses were conducted to examine the stability of the findings under different covariate specifications. In the expanded model, which additionally adjusted for department, weekly working days, professional title, position, and having children, empathy remained positively associated with psychological flexibility (path *a*: *B* = 0.093, SE = 0.034, β = 0.117, *p* = 0.007). Psychological flexibility also remained positively associated with flourishing after adjustment for empathy and the covariates (path *b*: *B* = 0.466, SE = 0.024, β = 0.622, *p* < 0.001). The total association between empathy and flourishing remained statistically significant (path *c*: *B* = 0.079, SE = 0.025, β = 0.133, *p* = 0.001), whereas the direct association did not reach statistical significance (path *c*′: *B* = 0.036, SE = 0.019, β = 0.060, *p* = 0.059). The estimated indirect association through psychological flexibility was 0.044 (BootSE = 0.017, 95% percentile bootstrap CI [0.010, 0.075]). The expanded outcome model explained 50.9% of the variance in flourishing (*R*^2^ = 0.509, adjusted *R*^2^ = 0.489).

In a further exploratory sensitivity analysis, concurrently measured job satisfaction was added to the expanded model. The associations corresponding to path a (*B* = 0.068, SE = 0.033, β = 0.086, *p* = 0.038) and path *b* (*B* = 0.403, SE = 0.023, β = 0.538, *p* < 0.001) remained statistically significant but were reduced in magnitude. The total association between empathy and flourishing remained statistically significant (path *c*: *B* = 0.053, SE = 0.022, β = 0.090, *p* = 0.016), whereas the direct association was not statistically significant (path *c*′: *B* = 0.026, SE = 0.018, β = 0.043, *p* = 0.145). The estimated indirect association through psychological flexibility was 0.028 (BootSE = 0.014, 95% percentile bootstrap CI [0.001, 0.054]). This outcome model explained 56.8% of the variance in flourishing (*R*^2^ = 0.568, adjusted *R*^2^ = 0.550).

Across all three models, the indirect association through psychological flexibility remained statistically significant. It was relatively stable after broader adjustment for occupational and family characteristics but was attenuated after concurrently measured job satisfaction was included. In contrast, the direct association between empathy and flourishing was small and sensitive to covariate specification, reaching statistical significance only in the primary model. Detailed regression results are presented in Supplementary Tables S1, S2, and comparisons across the three models are shown in Table 5.

**Table 5:** Comparison of the primary and sensitivity models for the indirect association between empathy and flourishing through psychological flexibility.

Association	Primary model	Expanded model	Expanded model with job satisfaction
Path *a*	0.098 (0.034); β = 0.124; *p* = 0.004	0.093 (0.034); β = 0.117; *p* = 0.007	0.068 (0.033); β = 0.086; *p* = 0.038
Path *b*	0.470 (0.023); β = 0.627; *p* < 0.001	0.466 (0.024); β = 0.622; *p* < 0.001	0.403 (0.023); β = 0.538; *p* < 0.001
Path *c*	0.086 (0.024); β = 0.144; *p* < 0.001	0.079 (0.025); β = 0.133; *p* = 0.001	0.053 (0.022); β = 0.090; *p* = 0.016
Path *c′*	0.039 (0.019); β = 0.066; *p* = 0.034	0.036 (0.019); β = 0.060; *p* = 0.059	0.026 (0.018); β = 0.043; *p* = 0.145
Indirect association	0.046 (0.016); Std. = 0.078; 95% CI [0.013, 0.078]	0.044 (0.017); Std. = 0.073; 95% CI [0.010, 0.075]	0.028 (0.014); Std. = 0.046; 95% CI [0.001, 0.054]

Values for paths *a, b, c*, and *c*′ are presented as *B* (SE), standardized coefficient β, and *p* value. Indirect associations are presented as *B* (BootSE), completely standardized indirect association (Std.), and 95% percentile bootstrap CI. Path a represents the association between empathy and psychological flexibility; path *b* represents the association between psychological flexibility and flourishing after adjustment for empathy; path *c* represents the total association between empathy and flourishing; and path *c*′ represents the direct association after psychological flexibility was included. Indirect associations were estimated using 5,000 percentile bootstrap samples. The primary model adjusted for age, gender, daily working hours, night shifts per month, marital status, and parental health status. The expanded model additionally adjusted for department, weekly working days, professional title, position, and having children. The exploratory model further included concurrently measured job satisfaction.

## 4 Discussion

### 4.1 Sociodemographic, occupational, and family factors associated with flourishing

In the adjusted model, age was positively associated with flourishing, whereas longer daily working hours, divorced marital status, and poorer parental health status were associated with lower flourishing. These findings suggest that nurses' flourishing may be related not only to individual psychological characteristics but also to occupational demands and family circumstances.

The positive association between age and flourishing may partly reflect the accumulation of clinical experience and coping resources over time, which may help nurses adapt to complex clinical situations and occupational stress and maintain positive psychological functioning (Beier et al., 2023; Schirda et al., 2016). In contrast, longer daily working hours may reduce opportunities for rest and recovery and increase nurses' physical and psychological burden, thereby making it more difficult to maintain positive psychological functioning (Ma, 2023; Che et al., 2023). This finding highlights the potential importance of reasonable working-hour arrangements and adequate recovery opportunities for nurses' mental health.

Lower flourishing among divorced nurses and nurses whose parents had poorer health may be related to changes in family relationships, caregiving responsibilities, and work–family demands. In the Chinese sociocultural context, parental health problems may place substantial emotional and caregiving responsibilities on adult children. When combined with demanding clinical work, these responsibilities may further deplete nurses' psychological resources (Yu et al., 2025; Yuan et al., 2023). Family circumstances and caregiving burden should therefore be considered alongside occupational stress when addressing nurses' positive mental health.

### 4.2 Associations of empathy and psychological flexibility with flourishing

The present study found that empathy was positively associated with flourishing, supporting the study hypothesis, although the magnitude of the association was weak. Previous studies have suggested that empathy may be associated with the professional meaning, interpersonal connection, and compassion satisfaction that healthcare professionals derive from patient care (Jiang et al., 2025; Higashibata et al., 2023; Milutinović et al., 2023). These positive professional experiences may partly explain the association observed between empathy and flourishing in the present study. However, the adjusted direct association between empathy and flourishing was small and did not reach statistical significance in the sensitivity models. This finding suggests that, although empathy is an important professional competency in nursing practice, its independent association with nurses' own flourishing may be limited and may be influenced by other personal and occupational factors.

In contrast, psychological flexibility showed a strong and relatively stable positive association with flourishing. In the hierarchical regression analysis, adding empathy increased the explained variance in flourishing by 2.0%, whereas the subsequent addition of psychological flexibility increased it by 37.7%. Previous studies have also shown that greater psychological flexibility is associated with better mental health and wellbeing (Sarabia-Cobo et al., 2021; Ramaci et al., 2019).

Psychological flexibility involves remaining open to difficult thoughts and emotions while continuing to act in accordance with personally meaningful values. Among clinical nurses, greater psychological flexibility may help them respond more flexibly to difficult internal experiences rather than avoiding or becoming overly entangled with them when facing patients' suffering, clinical demands, and their own emotional reactions, while continuing to engage in nursing behaviors that are consistent with professional values (Ebrahimi et al., 2024; Nicoletti et al., 2025). Therefore, how nurses respond to their internal experiences may provide an important psychological perspective for understanding their level of flourishing.

### 4.3 Indirect association through psychological flexibility

Empathy was positively but weakly associated with psychological flexibility, indicating that the two constructs are conceptually related but remain distinct. Empathic nursing care requires nurses to attend to patients' experiences while also noticing and responding to their own thoughts and emotions (Riess, 2017). Psychological flexibility reflects how nurses relate to these internal experiences and continue acting in accordance with professional values in complex situations (Sarabia-Cobo et al., 2021). This conceptual connection may help explain the observed indirect association.

The indirect association through psychological flexibility was statistically significant in the primary model and changed little after additional adjustment for department, weekly working days, professional title, position, and having children. This suggests that the observed association was relatively stable under broader adjustment for occupational and family characteristics. However, the indirect estimate was attenuated after concurrently measured job satisfaction was included. Job satisfaction was itself positively associated with both psychological flexibility and flourishing, suggesting that nurses' evaluations of their work may account for part of the shared associations among the study variables.

Because the present study used a cross-sectional design, the temporal ordering of empathy, psychological flexibility, and flourishing cannot be determined. Alternative explanations remain plausible. For example, greater flourishing may be associated with higher psychological flexibility and empathy, while psychological flexibility may facilitate empathic engagement rather than result from it. Personality traits, resilience, emotional intelligence, workplace climate, and organizational support may also influence all three constructs simultaneously. The findings should therefore be interpreted as a statistical indirect association consistent with the hypothesized model, rather than as evidence that psychological flexibility temporally or causally mediates the association between empathy and flourishing. Future studies using repeated longitudinal measurements are needed to compare alternative and reciprocal models, while intervention studies are required to determine whether changes in psychological flexibility precede subsequent changes in flourishing.

### 4.4 Clinical implications

The findings suggest that efforts to support nurses' flourishing should consider psychological resources, occupational demands, and family circumstances together. Nursing managers may pay particular attention to nurses with prolonged working hours or substantial family demands by improving workload arrangements, ensuring adequate rest and recovery, providing psychological support, and strengthening organizational support. The associations of job satisfaction with psychological flexibility and flourishing also indicate that working conditions, professional recognition, and managerial support may be important components of nurses' positive mental health.

The strong association between psychological flexibility and flourishing identifies psychological flexibility as a relevant factor for future occupational health research among nurses. ACT-related programs incorporating present moment awareness, acceptance, cognitive defusion, values clarification, and committed action may be considered as candidates for future intervention studies. However, the present study did not evaluate an intervention and cannot establish that increasing psychological flexibility will improve flourishing. Such programs should therefore be evaluated through longitudinal studies and randomized controlled trials.

Empathy remains fundamental to patient-centered care and effective nurse–patient relationships. Nevertheless, its weak association with nurses' flourishing suggests that empathy development should not be presented as a primary strategy for improving nurses' flourishing on the basis of the present findings. Future nurse-support programs may combine empathic competency development with psychological flexibility training and organizational-level improvements.

## 5 Limitations

This study has several limitations. The cross-sectional design precludes conclusions regarding temporal ordering or causality. Although the variables were ordered according to the hypothesized model, the statistical mediation analysis reflects indirect associations among concurrently measured variables and does not establish that psychological flexibility temporally or causally mediates the association between empathy and flourishing; alternative temporal orderings and reciprocal associations remain plausible. In addition, all main variables were assessed using self-report questionnaires administered through the same online survey, which may have introduced social desirability bias and common method variance. Harman's single-factor test provides only a preliminary assessment and cannot exclude the potential influence of common method bias. Despite covariate adjustment and sensitivity analyses, the possibility of residual confounding remains. The attenuation observed after job satisfaction was included suggests that the magnitude of some associations may partly depend on work-related factors, while unmeasured factors such as personality, resilience, emotional intelligence, workplace climate, and organizational support may also have contributed to the observed associations. Furthermore, participants were recruited through convenience sampling from three tertiary Grade A hospitals in Sichuan Province, and participation in the online survey was voluntary. This recruitment approach may have introduced selection bias, particularly self-selection bias. In addition, the sample consisted predominantly of female nurses with bachelor's degrees. The findings may therefore have limited generalizability to male nurses and nurses working in primary care, rural hospitals, private institutions, other regions, or healthcare systems outside China. The *post hoc* power analysis was based on the observed estimates and should be regarded as supplementary. Future studies should conduct an *a priori* power analysis based on expected indirect associations and adopt longitudinal, multicenter, and multisource designs.

## 6 Conclusion

This study identified positive cross-sectional associations among empathy, psychological flexibility, and flourishing in clinical nurses. Empathy was weakly associated with both psychological flexibility and flourishing, whereas psychological flexibility showed a substantially stronger and relatively stable association with flourishing. The indirect association between empathy and flourishing through psychological flexibility was statistically significant and remained similar after broader adjustment for occupational and family characteristics, although it was attenuated after concurrently measured job satisfaction was included. These findings highlight psychological flexibility as an important correlate of nurses' flourishing, but they do not establish temporal ordering or causal mediation. Future longitudinal and intervention studies are needed to clarify the direction of these associations and to determine whether psychological flexibility-related and organizational strategies can support nurses' flourishing.

## Data Availability

The raw data supporting the conclusions of this article will be made available by the authors, without undue reservation.
