# Supplementary material for: The role of psychological flexibility in the relationship between empathy and flourishing among clinical nurses: a cross-sectional mediation analysis
Source: Front Psychol. 2026 Sep 3;17:1923032. doi: 10.3389/fpsyg.2026.1923032 (PMC13581948; doi:10.3389/fpsyg.2026.1923032)
Supplement: Supplementary file 1 [file Table_1.docx]

**Supplementary Table S1. Expanded Hierarchical Multiple Linear Regression Analysis of Variables Associated With Flourishing**

| **Variables** | **Model 1 B (SE)** | **β** | ***p*** | **Model 2 B (SE)** | **β** | ***p*** | **Model 3 B (SE)** | **β** | ***p*** |
| --- | --- | --- | --- | --- | --- | --- | --- | --- | --- |
| **Constant** | 57.095 (7.582) | - | <0.001 | 47.400 (8.097) | - | <0.001 | 23.236 (6.266) | - | <0.001 |
| **Age** | 0.081 (0.076) | 0.072 | 0.289 | 0.078 (0.076) | 0.070 | 0.300 | 0.144 (0.057) | 0.128 | 0.012 |
| **Gender** |  |  |  |  |  |  |  |  |  |
| Female | 0.412 (1.353) | 0.013 | 0.761 | 0.211 (1.343) | 0.007 | 0.875 | −0.465 (1.020) | −0.015 | 0.648 |
| **Department** |  |  |  |  |  |  |  |  |  |
| Outpatient | 1.457 (1.334) | 0.051 | 0.276 | 1.448 (1.323) | 0.050 | 0.274 | 0.759 (1.004) | 0.026 | 0.450 |
| Surgery | 0.446 (0.856) | 0.025 | 0.602 | 0.621 (0.850) | 0.034 | 0.465 | 0.189 (0.645) | 0.010 | 0.770 |
| Emergency/ICU | 3.170 (1.506) | 0.104 | 0.036 | 3.222 (1.493) | 0.106 | 0.031 | 1.441 (1.136) | 0.047 | 0.205 |
| Other Departments | 2.475 (0.977) | 0.120 | 0.012 | 2.305 (0.969) | 0.112 | 0.018 | 1.533 (0.737) | 0.074 | 0.038 |
| **Daily Working Hours** | −1.369 (0.380) | −0.162 | <0.001 | −1.266 (0.378) | −0.150 | 0.001 | −0.766 (0.288) | −0.090 | 0.008 |
| **Weekly Working Days** | −0.498 (1.133) | −0.019 | 0.661 | −0.273 (1.125) | −0.011 | 0.808 | −0.927 (0.855) | −0.036 | 0.279 |
| **Night Shifts per Month** | −0.058 (0.096) | −0.030 | 0.546 | −0.072 (0.096) | −0.037 | 0.452 | −0.002 (0.073) | −0.001 | 0.975 |
| **Position** |  |  |  |  |  |  |  |  |  |
| Nursing Educator | 0.815 (0.996) | 0.036 | 0.414 | 0.603 (0.990) | 0.027 | 0.542 | 0.254 (0.751) | 0.011 | 0.736 |
| Nurse Manager | 3.537 (1.768) | 0.112 | 0.046 | 3.194 (1.756) | 0.102 | 0.069 | 0.751 (1.338) | 0.024 | 0.575 |
| **Marital Status** |  |  |  |  |  |  |  |  |  |
| Unmarried | −0.181 (1.223) | −0.010 | 0.883 | 0.067 (1.215) | 0.004 | 0.956 | 0.785 (0.923) | 0.041 | 0.395 |
| Divorced | −7.796 (2.087) | −0.155 | <0.001 | −7.688 (2.069) | −0.153 | <0.001 | −6.048 (1.573) | −0.121 | <0.001 |
| **Having Children** |  |  |  |  |  |  |  |  |  |
| Yes | 0.995 (1.187) | 0.058 | 0.402 | 1.169 (1.178) | 0.068 | 0.321 | 0.962 (0.894) | 0.056 | 0.282 |
| **Parental Health Status** |  |  |  |  |  |  |  |  |  |
| Chronic/Major Illness | −3.596 (0.862) | −0.191 | <0.001 | −3.552 (0.854) | −0.188 | <0.001 | −2.612 (0.650) | −0.138 | <0.001 |
| Suboptimal Health | −1.946 (0.811) | −0.108 | 0.017 | −2.127 (0.806) | −0.118 | 0.009 | −1.342 (0.613) | −0.074 | 0.029 |
| Other | −7.810 (2.396) | −0.140 | 0.001 | −7.902 (2.375) | −0.141 | 0.001 | −7.323 (1.802) | −0.131 | <0.001 |
| **Professional Title** |  |  |  |  |  |  |  |  |  |
| Intermediate Title | 0.345 (1.001) | 0.021 | 0.730 | 0.241 (0.993) | 0.015 | 0.808 | 0.204 (0.754) | 0.012 | 0.786 |
| Senior Title | −0.397 (2.251) | −0.011 | 0.860 | −0.401 (2.231) | −0.011 | 0.857 | −0.568 (1.693) | −0.016 | 0.737 |
| **Empathy** | - | - | - | 0.079 (0.025) | 0.133 | 0.001 | 0.036 (0.019) | 0.060 | 0.059 |
| **Psychological Flexibility** | - | - | - | - | - | - | 0.466 (0.024) | 0.622 | <0.001 |
| *R*² | 0.128 |  |  | 0.145 |  |  | 0.509 |  |  |
| Adjusted *R*² | 0.097 |  |  | 0.113 |  |  | 0.489 |  |  |
| Δ*R*² | 0.128 |  |  | 0.017 |  |  | 0.363 |  |  |
| *F* | 4.067 |  | <0.001 | 4.451 |  | <0.001 | 25.784 |  | <0.001 |
| Δ*F* | 4.067 |  | <0.001 | 10.365 |  | 0.001 | 386.892 |  | <0.001 |

**Note.** B=unstandardized regression coefficient; SE=standard error; β=standardized regression coefficient. Model 1 included Age, Gender, Department, Daily Working Hours, Weekly Working Days, Night Shifts per Month, Position, Marital Status, Having children, Parental Health Status, and Professional Title. Model 2 additionally included Empathy, and Model 3 additionally included Psychological Flexibility. Age, Daily Working Hours, Weekly Working Days, and Night Shifts per Month were entered as continuous variables using their original values. Categorical covariates were dummy coded. The reference categories were Male, Internal Medicine, Staff Nurse, Married, Healthy, Junior title, and No children for Gender, Department, Position, Marital Status, Parental Health Status, Professional Title, and Having children, respectively. Professional Title was recategorized as Junior title (Nurse and Senior Nurse), Intermediate title (Nurse-in-Charge), and Senior title (Associate Chief Nurse and Chief Nurse) before dummy coding. The Durbin–Watson statistic for Model 3 was 2.167, and all variance inflation factors were below 3.

**Supplementary Table S2. Exploratory Hierarchical Multiple Linear Regression Analysis of Variables Associated With Flourishing After Additional Adjustment for Job Satisfaction**

| **Variables** | **Model 1 B (SE)** | **β** | ***p*** | **Model 2 B (SE)** | **β** | ***p*** | **Model 3 B (SE)** | **β** | ***p*** |
| --- | --- | --- | --- | --- | --- | --- | --- | --- | --- |
| **Constant** | 35.046 (6.970) | - | <0.001 | 28.957 (7.378) | - | <0.001 | 15.266 (5.954) | - | 0.011 |
| **Age** | 0.071 (0.068) | 0.063 | 0.295 | 0.069 (0.067) | 0.062 | 0.304 | 0.130 (0.054) | 0.115 | 0.016 |
| **Gender** |  |  |  |  |  |  |  |  |  |
| Female | 0.710 (1.200) | 0.023 | 0.555 | 0.568 (1.196) | 0.018 | 0.635 | −0.156 (0.957) | −0.005 | 0.870 |
| **Department** |  |  |  |  |  |  |  |  |  |
| Outpatient | 0.028 (1.189) | 0.001 | 0.981 | 0.052 (1.184) | 0.002 | 0.965 | 0.002 (0.947) | 0.000 | 0.999 |
| Surgery | 0.595 (0.759) | 0.033 | 0.433 | 0.710 (0.757) | 0.039 | 0.348 | 0.301 (0.606) | 0.017 | 0.619 |
| Emergency/ICU | 1.606 (1.342) | 0.053 | 0.232 | 1.673 (1.336) | 0.055 | 0.211 | 0.738 (1.070) | 0.024 | 0.491 |
| Other Departments | 1.517 (0.870) | 0.073 | 0.082 | 1.422 (0.866) | 0.069 | 0.101 | 1.099 (0.693) | 0.053 | 0.113 |
| **Daily Working Hours** | −0.888 (0.339) | −0.105 | 0.009 | −0.828 (0.338) | −0.098 | 0.015 | −0.567 (0.271) | −0.067 | 0.037 |
| **Weekly Working Days** | −0.394 (1.005) | −0.015 | 0.695 | −0.245 (1.002) | −0.010 | 0.807 | −0.821 (0.802) | −0.032 | 0.306 |
| **Night Shifts per Month** | 0.034 (0.086) | 0.017 | 0.694 | 0.023 (0.086) | 0.012 | 0.791 | 0.046 (0.068) | 0.023 | 0.502 |
| **Position** |  |  |  |  |  |  |  |  |  |
| Nursing Educator | 0.836 (0.883) | 0.037 | 0.345 | 0.693 (0.881) | 0.031 | 0.432 | 0.355 (0.705) | 0.016 | 0.614 |
| Nurse Manager | 1.582 (1.576) | 0.050 | 0.316 | 1.391 (1.571) | 0.044 | 0.376 | −0.017 (1.259) | −0.001 | 0.989 |
| **Marital Status** |  |  |  |  |  |  |  |  |  |
| Unmarried | −0.488 (1.085) | −0.026 | 0.653 | −0.315 (1.082) | −0.017 | 0.771 | 0.456 (0.867) | 0.024 | 0.599 |
| Divorced | −7.237 (1.851) | −0.144 | <0.001 | −7.175 (1.843) | −0.143 | <0.001 | −5.958 (1.476) | −0.119 | <0.001 |
| **Having Children** |  |  |  |  |  |  |  |  |  |
| Yes | −0.251 (1.057) | −0.015 | 0.813 | −0.109 (1.054) | −0.006 | 0.918 | 0.212 (0.843) | 0.012 | 0.802 |
| **Parental Health Status** |  |  |  |  |  |  |  |  |  |
| Chronic/Major Illness | −2.851 (0.767) | −0.151 | <0.001 | −2.837 (0.763) | −0.150 | <0.001 | −2.303 (0.611) | −0.122 | <0.001 |
| Suboptimal Health | −1.333 (0.721) | −0.074 | 0.065 | −1.467 (0.720) | −0.081 | 0.042 | −1.046 (0.576) | −0.058 | 0.070 |
| Other | −6.105 (2.129) | −0.109 | 0.004 | −6.201 (2.120) | −0.111 | 0.004 | −6.365 (1.695) | −0.114 | <0.001 |
| **Professional title** |  |  |  |  |  |  |  |  |  |
| Intermediate Title | 0.900 (0.889) | 0.055 | 0.312 | 0.819 (0.885) | 0.050 | 0.355 | 0.561 (0.708) | 0.034 | 0.428 |
| Senior Title | 0.618 (1.997) | 0.018 | 0.757 | 0.595 (1.988) | 0.017 | 0.765 | 0.061 (1.590) | 0.002 | 0.970 |
| **Job Satisfaction** | 4.691 (0.391) | 0.458 | <0.001 | 4.597 (0.391) | 0.449 | <0.001 | 2.800 (0.330) | 0.273 | <0.001 |
| **Empathy** | - | - | - | 0.053 (0.022) | 0.090 | 0.016 | 0.026 (0.018) | 0.043 | 0.145 |
| **Psychological Flexibility** | - | - | - | - | - | - | 0.403 (0.023) | 0.538 | <0.001 |
| *R*² | 0.316 |  |  | 0.324 |  |  | 0.568 |  |  |
| Adjusted *R*² | 0.290 |  |  | 0.296 |  |  | 0.550 |  |  |
| Δ*R*² | 0.316 |  |  | 0.008 |  |  | 0.245 |  |  |
| *F* | 12.104 |  | <0.001 | 11.916 |  | <0.001 | 31.227 |  | <0.001 |
| Δ*F* | 12.104 |  | <0.001 | 5.881 |  | 0.016 | 295.749 |  | <0.001 |

**Note.** B=unstandardized regression coefficient; SE=standard error; β=standardized regression coefficient. Model 1 included Age, Gender, Department, Daily Working Hours, Weekly Working Days, Night Shifts per Month, Position, Marital Status, Having children, Parental Health Status, Professional Title, and Job Satisfaction. Model 2 additionally included Empathy, and Model 3 additionally included Psychological Flexibility. Age, Daily Working Hours, Weekly Working Days, and Night Shifts per Month were entered as continuous variables using their original values. Job Satisfaction was entered using its original five-point score. Categorical covariates were dummy coded. The reference categories were Male, Internal Medicine, Staff Nurse, Married, Healthy, Junior title, and No children for Gender, Department, Position, Marital Status, Parental Health Status, Professional Title, and Having children, respectively. Professional Title was recategorized as Junior title (Nurse and Senior Nurse), Intermediate title (Nurse-in-Charge), and Senior title (Associate Chief Nurse and Chief Nurse) before dummy coding. Because Job Satisfaction was measured concurrently with Empathy, Psychological Flexibility, and Flourishing, this model was regarded as an exploratory sensitivity analysis. The Durbin–Watson statistic for Model 3 was 2.118, and all variance inflation factors were below 3.
